# Rhabdoid Tumor Predisposition Syndrome: From Clinical Suspicion to General Management

**DOI:** 10.3389/fonc.2021.586288

**Published:** 2021-02-22

**Authors:** Giada Del Baldo, Roberto Carta, Iside Alessi, Pietro Merli, Emanuele Agolini, Martina Rinelli, Luigi Boccuto, Giuseppe Maria Milano, Annalisa Serra, Andrea Carai, Franco Locatelli, Angela Mastronuzzi

**Affiliations:** ^1^ Department of Paediatric Haematology/Oncology, IRCCS Bambino Gesù Children’s Hospital, Rome, Italy; ^2^ Laboratory of Medical Genetics, IRCCS Bambino Gesù Children’s Hospital, Rome, Italy; ^3^ JC Self Research Institute, Greenwood Genetic Center, Greenwood, SC, United States; ^4^ School of Nursing, College of Behavioral, Social and Health Science, Clemson University, Clemson, SC, United States; ^5^ Department of Neuroscience and Neurorehabilitation, Neurosurgery Unit, IRCCS Bambino Gesù Children’s Hospital, Rome, Italy; ^6^ Department of Maternal, Infantile, and Urological Sciences, University of Rome La Sapienza, Rome, Italy

**Keywords:** rhabdoid tumors, atypical teratoid/rhabdoid tumors, cancer surveillance, genetic test, cancer risk, cancer predisposition syndromes

## Abstract

Rhabdoid tumors are rare aggressive malignancies in infants and young children with a poor prognosis. The most common anatomic localizations are the central nervous system, the kidneys, and other soft tissues. Rhabdoid tumors share germline and somatic mutations in *SMARCB1* or, more rarely, *SMARCA4*, members of the SWI/SNF chromatin-remodeling complex. Rhabdoid tumor predisposition syndrome (RTPS) is a condition characterized by a high risk of developing rhabdoid tumors, among other features. RTPS1 is characterized by pathogenic variants in the *SMARCB1* gene, while RTPS2 has variants in *SMARCA4*. Interestingly, germline variants of *SMARCB1* and *SMARCA4* have been identified also in patients with Coffin-Siris syndrome. Children with RTPS typically present with tumors before 1 year of age and in a high percentage of cases develop synchronous or multifocal tumors with aggressive clinical features. The diagnosis of RTPS should be considered in patients with rhabdoid tumors, especially if they have multiple primary tumors and/or in individuals with a family history. Because germline mutations result in an increased risk of carriers developing rhabdoid tumors, genetic counseling, and surveillance for all family members with this condition is recommended.

## Introduction

Rhabdoid tumor predisposition syndrome (RTPS) is characterized by an elevated risk of developing malignancies called rhabdoid tumors (RTs). RTs are rare, aggressive tumors, typically diagnosed in infants (1).

Primary rhabdoid tumor sites can include the central nervous system (65%), kidney (9%) and in the remaining 26% of cases: head and neck soft tissues, paravertebral muscles, liver, bladder, mediastinum, retroperitoneum, and pelvis (2).

Immunohistochemical characteristics of these tumors include loss of the BAF47/BRG1 protein (3). Among newly diagnosed cases, 25%–35% will harbor a germline variant of the *SMARCB1* gene (OMIM*601607) (4, 5). Recently, pathogenic variants in the *SMARCA4* gene (OMIM*603254) have also been associated with RT (6); while the involvement of other genes appears to be exceedingly rare in RTs (7, 8).

The most frequent pediatric tumor associated with RTPS is atypical teratoid/rhabdoid tumor (AT/RT). AT/RTs are rare, accounting for 1%–2% of all brain cancers, 90% of cases being diagnosed in children of less than 3 years of age (9–12), with a slight male predominance (13). At the time of presentation, 65.4% are in the posterior fossa, 31% supratentorial and 3.6% multifocal (14).

Histologically, AT/RT shows areas of rhabdoid phenotype containing rhabdoid cells with eccentric nuclei, prominent nucleoli, abundant eosinophilic cytoplasm, and a mesenchymal component with spindle cells. In the last years, molecular characterization of RT has become increasingly relevant. *SMARCB1* and *SMARCA4* are tumor suppressor genes playing a critical etiologic role in all rhabdoid tumors including AT/RT, which is linked to somatic and germline mutations of *SMARCB1* or, more rarely, *SMARCA4*.

AT/RTs are biologically heterogeneous. In the last few years, different authors described transcriptional features of AT/RTs that can be summarized in three molecular subgroups (12, 15–17) with different genetic profile, age at onset, prognosis, and brain localization:

1) AT/RT-TYR tumors are characterized by infratentorial location, younger age at diagnosis (<1 year) and overexpression of the melanosomal markers such as DCT, TYR, and MITF and many genes involved in ciliogenesis (*DNAH11* and *SPEF1*). Other pathways described include bone morphogenetic protein (BMP) and orthodenticle homeobox 2 (OTX2). Chromosome 22q loss is the most common cytogenetic anomaly.
2) AT/RT-MYC tumors are generally supratentorial, affected individuals are older (age 4–5 years), and the cluster genes *MYC*, *HOTAIR*, and *HOX* are overexpressed. Focal deletions of *SMARCB1* are the most common molecular anomaly. Supratentorial location is the more frequent site. Spinal tumors are included in this subgroup.
3) AT/RT-SHH tumors location may be infratentorial or supratentorial with similar frequency, diagnosis is in the age interval 2 to 5 years. Genes of the sonic hedgehog pathway (*GLI2*, *BOC*, *PTCHD2*) and NOTCH signaling (*ASLC1*, *CBL*, *HES1*) are overexpressed.

Patients outcome for each group is not homogeneous among the different data published to date and prognosis is still unclear (12, 15–17).

The most common extra-cerebral site for the primary onset of an RT is the kidney (48% of cases), followed by head and neck (14%), liver (13%), and other sites such as trunk and arms (25%) (18, 19).

RTs of the kidney account for about 2% of all pediatric renal cancers (20). Renal RT is highly aggressive and has a poor prognosis, with a 12-month survival rate of only 30% (18). Patients presenting with renal RT in the first year of life tend to develop brain tumors in 10%–15% of cases (21). These patients often harbor a germline mutation of *SMARCB1* and have a worse prognosis, as compared to those with sporadic RTs (22).

## Rhabdoid Tumor Predisposition Syndrome

RTPS is an autosomal dominant cancer predisposition syndrome. When the mutation pathogenic variants occur in the *SMARCB1* gene, the syndrome is called RTPS1, and RTPS2 has variants in the *SMARCA4* gene.

BAF47/BRG1 proteins encoded by *SMARCB1/SMARCA4* genes are key components of the ATP-dependent chromatin-remodeling SWI/SNF complex, which is essential for lineage specification, gene regulation, and maintenance of stem cell pluripotency (23).

RTs are the most frequent malignancies associated with these syndromes, but not the only ones. In most cases these arise *de novo* but there is a small percentage of familial cases having RTPS. RTs can present in a familial setting, with up to 35% of cases due to germline mutations in *SMARCB1* (4) or, in 2%–3% of cases, in *SMARCA4* (24, 25).

Children with RTPS typically present with tumors before 12 months of age and in 35% of cases develop synchronous or multifocal tumors with aggressive clinical features (20, 22, 26). RTs can be detected in the prenatal period or during childhood with a median age at onset of 4–7 months (range prenatally – 60 months) (1, 27, 28) versus sporadic RTs that are detected at a median age of 13–30 months (range: age 1 day–228 months). Often RTs in RTPS are synchronous, with advanced stage at diagnosis and clinically aggressive. Progression occurs during chemotherapy in 58% of individuals with RTPS and RTs (24). In the EU-RHAB Registry 28% of cases had synchronous RT: eight individuals AT/RT and extracranial malignant rhabdoid tumors (eMRT), four had AT/RT and rhabdoid tumor of the kidney (RTK), and two AT/RT, multiple eMRT and RTK (28).

Furthermore, other conditions are known to be related to RTPS. Family history of RT or cribriform neuroepithelial tumor (CRINET) and/or combination of RT with one of the following: schwannoma, malignant peripheral nerve sheath tumor, meningioma are highly suggestive for RTPS (29).

**Figure 1:**
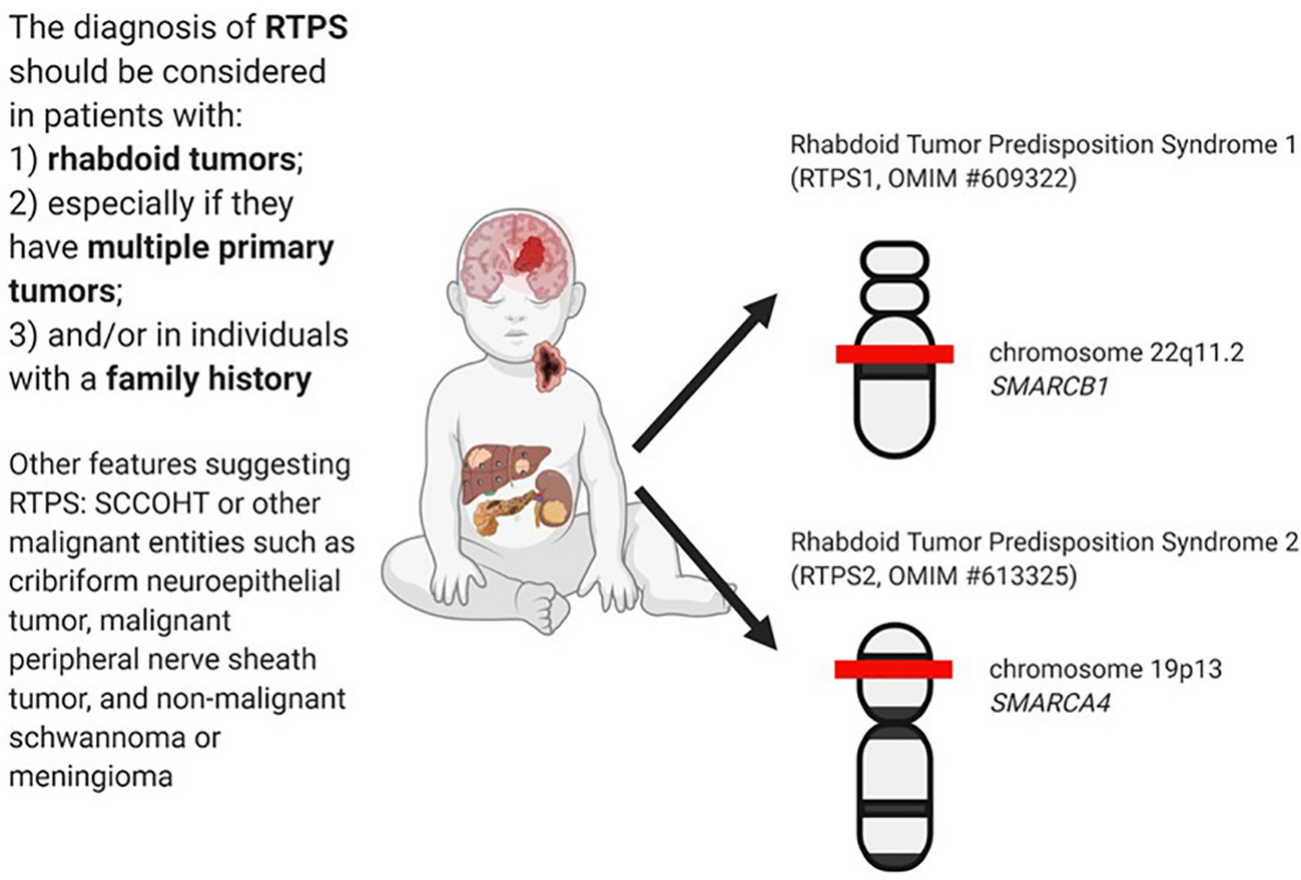
RTPS tumors spectrum and related genes involved.

The diagnosis of RTPS is established in a proband with a rhabdoid tumor and/or a family history of RT and/or multiple SMARCB1/SMARCA4 deficient tumors (synchronous or metachronous) and identification of a germline pathogenic variant in *SMARCB1* or *SMARCA4* by genetic testing (30). In **Figure 1** are summarized the main clinical and genetics features of RTPS.

### Rhabdoid Tumor Predisposition Syndrome 1

Rhabdoid Tumor Predisposition Syndrome 1 (RTPS1, OMIM #609322) is caused by heterozygous germline mutations in the *SMARCB1* gene, which maps to chromosome 22q11.2 (31). The protein involved is an SWI/SNF-related matrix-associated actin-dependent regulator of chromatin subfamily B member 1 (30).

#### Clinical Features

As described above, the syndrome predisposes to the development of RTs, including brain tumors, renal and extrarenal cancers. AT/RT is the most frequent brain cancer in patients with *SMARCB1* mutations, but other CNS tumors are described (32).

Interestingly, Thomas et al. (33) described a case of RTPS1 in an infant with AT/RT in which supratentorial and infratentorial parts of the tumor demonstrated different DNA methylation profiles suggesting synchronous or metachronous AT/RT with different molecular subgroup and cell of origin.

Recently, the *SMARCB1* gene has been found also in familial and sporadic schwannomatosis. Hulsebos et al. (34) described two family members with schwannomatosis and a germline mutation of *SMARCB1*, suggesting it as a candidate predisposing gene. Swensen et al. reported a family with hereditary schwannomatosis associated with a germline mutation of *SMARCB1*. Three members of the family developed RTs and died before 2 years of age (35). About 40%–50% of familial schwannomatosis and 8%–10% of sporadic cases harbor a constitutional mutation in *SMARCB1* (25). Interestingly, *SMARCB1* and *NF2* loci map very close to each other on the long arm of chromosome 22 (25).

Furthermore, Schmitz et al. found the same somatic mutation of *SMARCB1* in four of 126 meningiomas. The data suggest that *SMARCB1* is a tumor suppressor gene that may be important also for the oncogenesis in a subset of meningiomas (36).

Moreover, *SMARCB1* mutation carriers may be at risk for developing other tumors such as malignant peripheral nerve sheath tumors and cribriform neuroepithelial tumors (37).

#### Genetics

*SMARCB1* inactivation can be caused by different mechanisms like gross chromosomal aberration or loss of heterozygosity of 22q11.2 or loss-of-function mutations including nonsense, frameshift, splicing and missense mutations (6).

Concerning cytogenetics, the most frequent alteration described in AT/RT is the monosomy of chromosome 22 (14, 38, 39). Biegel et al. described also a rhabdoid tumor with an unbalanced 9;22 translocation (40).

*Penetrance*. Penetrance may vary according to the mutation type. Incomplete penetrance has been observed in three of nine published families with RTPS due to *SMARCB1* mutations (6). Rarely a SMARCB1  pathogenic variant is inherited from an unaffected parent or a parent with late-onset or undiagnosed RTPS (41). Germline  mosaicism must be taken into account for at least half of the families with sibs  affected by RTPS (30).

### Rhabdoid Tumor Predisposition Syndrome 2

Rhabdoid Tumor Predisposition Syndrome 2 (RTPS2, OMIM #613325) is caused by heterozygous germline mutations in the *SMARCA4* gene, which maps to chromosome 19p13 (6) and encodes a protein involved in the transcription activator BRG1, a catalytic component of the ATP-dependent SWI/SNF chromatin remodeling complex (30).

#### Clinical Features

The main tumor resulting from germline pathogenic variants in *SMARCA4* is small cell carcinoma of the ovary, hypercalcemic type (SCCOHT) (37, 42). It seems that up to 40% of females with SCCOHT may harbor a germline variant in *SMARCA4* (43), therefore the detection of SCCOHT in young women is high evocative for RTPS2 (44–46).

Although more rarely than *SMARCB1* mutations, pathogenic germline *SMARCA4* variants are found in children with AT/RT and it seems that *SMARCA4*-mutated AT/RT may be associated with a worse prognosis (24, 47). The risk of other RTs in *SMARCA4* germline heterozygotes is unknown, but probably very low.

Other epithelial cancers, such as lung cancer, have been reported in some adults with pathogenic germline variants in *SMARCA4*, but again, the risks remain unquantified (46).

Recently, a novel entity designated “*SMARCA4*-deficient thoracic sarcoma” (SDTS) was described by Le Loarer et al. in 19 adult individuals, supporting the carcinogenic effect of *SMARCA4* inactivation, with consequences beyond the pediatric age range (48).

#### Genetics

Among the different *SMARCA4* pathogenic variants reported to date, nonsense, and intragenic deletions are the prevalent types, while only a single missense variant has been detected (24).

*Penetrance.* It appears that *SMARCA4* mutations are less penetrant for AT/RT than *SMARCB1* ones (37). In contrast to *SMARCB1*, most reported patients with RTs and a *SMARCA4* mutation inherited it from an unaffected parent (30). In *SMARCA4*-related RTPS, the penetrance for RT in the preceding generation of seven informative families was zero. However, in one family, two sibs with a SMARCA4 pathogenic variant were both affected (6, 24, 30).

### Other Rare Manifestations Related to SMARCB1 and SMARCA4 Mutations

Interestingly, germline variants of *SMARCB1* and *SMARCA4* have been identified also in patients with Coffin-Siris syndrome three (CSS3, OMIM #614608) and four (CSS4, OMIM #614609). CSS is a congenital malformation syndrome characterized by developmental delay, intellectual disability, coarse facial features, feeding difficulties, and hypoplastic or absent fifth fingernails and fifth distal phalanges (49). Individuals with CSS carrying *SMARCB1* or *SMARCA4* mutations seem to show no predisposition to develop RTs or other forms of tumor. This can be explained by the fact that mutations resulting in CSS3 are non-truncating, implying that they exert gain-of-function or dominant-negative effects (excluding haploinsufficiency as a cause) (50). Very rare exceptions have been described. To date, a single CSS individual with schwannomatosis and a *SMARCB1* variant has been reported (51): the *SMARCB1* c.1121G>A(p.Arg374Gln) germline transition in exon 9 lead to the inactivation of the second allele in the tumor tissue. More recently, a pediatric patient with mild CSS who concomitantly developed small‐cell carcinoma of the ovary hypercalcaemic type has been found to harbor a germline heterozygous nonsense mutation and a somatic frameshift mutation in *SMARCA4* (52).

## Genotype-Phenotype Correlation

According to Smith et al. and Holsten et al. a clear genotype-phenotype correlation could be identified (53, 54). Germline *SMARCB1* mutations located in the central portion of the gene, involving multiple exon deletions or duplications and truncating mutations, likely responsible for a loss of SMARCB1 protein product, are most frequently associated with rhabdoid tumors. Instead, *SMARCB1* mutations located at the ends of the gene, particularly non-truncating alterations, including missense variants, are most frequently associated with non-oncologic diseases and low-grade tumors such as the ones reported in CSS, meningiomas, and schwannomas. Unlike the germline *SMARCB1* mutations detected in RT cases, schwannomatosis-associated alterations determine reduced expression levels or a partial loss of function of the SMARCB1 protein (53). Moreover, a correlation was identified between the type of *SMARCB1* variant and the time of onset of the disease: truncating variants are associated with early-onset disease, non-truncating variants with late-onset disease.

## Surveillance

To date, no universally accepted surveillance recommendations for RTPS carriers have been established. In **Table 1** are summarized two surveillance propositions suggested by Foulkes et al. (37) and Teplick et al. (55). Nemes et al. (30) proposed a protocol of surveillance not only in pre-symptomatic RTPS carriers but also in individuals affected by RTs.

**Table 1:** Surveillance recommendations for rhabdoid tumor predisposition syndrome (RTPS) carriers.

Foulkes et al. (37)	Teplick et al. (55)
***Germline truncating mutations:*** *SMARCB1* - Brain: MRI every 3 months to age 5 years - Abdomen: Ultrasound every 3 months through 5 years. Consider WB-MRI, undetermined frequency *SMARCA4* - Brain: No data available, risks likely very low - Abdomen: No data available, risk likely low to very low - Ovary: No data available, abdominal ultrasound every 6 months may be justified, role, if any, of MRI unknown. Preventive oophorectomy may be justified outside of the pediatric age range ***Germline missense mutations:*** No screening, generally no/very low risk	- From 0–1 year: is recommended abdominal US every 2 to 3 months and head US monthly - From 1–4 years: abdominal US every 6 months. Brain and spine MRI every 6 months

MRI, magnetic resonance imaging; WB-MRI, whole body magnetic resonance imaging; US, ultrasound.

Foulks et al. (37) give more detailed indications about monitoring of *SMARCB1* or *SMARCA4* carriers as opposed to Teplick et al. (55), even if they failed to stratify cancer monitoring for age range. They recommended brain MRI in *SMARCB1* carriers every 3 months for the first 5 years of life. As known, AT/RTs in RTPS1 arise generally within the first year of life and MRI is an expensive examen, and sedation is needed in young children. After the first year of life, a brain MRI should be performed every 6 months. About abdominal monitoring, they recommended ultrasound every 3 months through 5 years and consider whole-body MRI, with undetermined frequency. Whole-body MRI will guarantee high diagnostic accuracy as opposed to ultrasound, but it is an expensive procedure and requires sedation in little patients.

Regarding *SMARCA4* carriers they suggest an abdominal ultrasound every 6 months with no mention of the beginning or end of the follow-up. Considering the rarity of the condition and the very low risk, unfortunately, there is no data available for monitoring of brain and abdominal RTs in *SMARCA4* carriers.

Interestingly, Folkes et al. (37) proposed a separated surveillance protocol for germline truncating mutations versus germline missense mutations, underlining that germline missense mutations need no screening for their very low risk of RTs. On the other hand, they proposed MRI surveillance for patients with a germline missense mutation of *SMARCB1* to allow the early detection of schwannomas.

Teplick et al. (55) did not take into account the due separated conditions RTPS1 and 2 and different germline kinds of mutations. They suggested the use of ultrasound in the first year of life to monitor the brain and abdomen every 2–3 months. Between 1 and 4 years of age, they suggest extending abdominal ultrasound monitoring every 6 months and using brain and spine MRI to exclude the onset of brain tumors every 6 months. In their proposal, there is no mention of whole-body MRI.

## Genetic Test

Molecular genetic testing for RTPS is appropriate in any patients with:

- RTs, familial RTs, multifocal or synchronous tumor, congenital or early-onset disease, other conditions known to be related to RTPS
- *SMARCB1*- or *SMARCA4*-deficient tumors with a positive family history.

Point variants of *SMARCB1* and *SMARCA4* can be identified by Sanger sequencing or next-generation sequencing (NGS). Besides point mutations, other alterations of *SMARCB1* and *SMARCA4* have also been documented, including deletion of the entire *SMARCB1* locus or intragenic deletions involving one or more exons (5). Methods used to detect this kind of alteration may include quantitative PCR, multiplex ligation-dependent probe amplification (MLPA), and a gene-targeted microarray designed to detect single-exon deletions or duplications.

## Genetic Counseling and Risk to Family Members

### Siblings and Parents

When a pathogenic variant of *SMARCB1* or *SMARCA4* is detected in a proband, molecular genetic evaluation of parents and siblings is required.

As mentioned above, carriers of *SMARCA4* mutation inherited a pathogenic variant from an unaffected parent (24), while the vast majority of individuals with RTPS1 have a *de novo* germline *SMARCB1* mutation, and only in extremely rare cases, they inherited a *SMARCB1* pathogenic variant from an unaffected parent.

A healthy parent with a pathogenic germline variant has to start surveillance as for siblings, but at longer intervals, as the risk of malignancies is very low.

If the *SMARCA4* or *SMARCB1* pathogenic variant found in the proband cannot be detected in either parent, it raises the possibility of a *de novo* pathogenic variant in the proband or germline mosaicism in a parent. Parental germline mosaicism in *SMARCB1* has been rarely described (5, 27, 32, 56, 57), while the overall incidence of germline mosaicism in RTPS is unknown.

The cancer risk for the siblings of a proband depends on the genetic status of the proband’s parents:

- 50% risk of inheriting the variant if the proband harbors a *SMARCA4* or *SMARCB1* pathogenic variant, although penetrance can be incomplete.
- 1% risk of inheriting the variant if the parent is negative for *SMARCA4* or *SMARCB1* mutations, considering the possibility of parental germline mosaicism (5, 27, 56, 57).

### Offspring of a Proband

As mentioned above, patients with RTPS1 die at a young age. Despite it occurs very rarely, it should be considered the cancer risk in offsprings. If children are affected by a *de novo* germline *SMARCB1* mutation and survive to adulthood, they can potentially transmit the mutation to their offspring (25).

The family history of most individuals with RTPS may appear to be negative for many reasons: failure of detection of the disorder in family members, reduced penetrance (more evident in *SMARCA4*-related RTPS), late onset in the affected parent.

## Prevention and Prenatal Diagnosis

There is no possibility of preventing cancer development in patients with RTPS, but in case of detected *SMARCB1*/*SMARCA4* mutations, the advice of surveillance and follow-up must be followed. Prophylactic oophorectomy may be discussed in women with *SMARCA4*-related RTPS for the high risk to develop SCCOHT (58).

It would also be important to prevent secondary complications related to aggressive treatments.

Once *SMARCB1* and *SMARCA4* pathogenic variants are detected, prenatal testing for a pregnancy at increased risk and preimplantation genetic diagnosis are possible. The preferred tests used to assess if a product of conception carries a known *SMARCB1*/*SMARCA4* mutation are chorionic villus sampling and amniocentesis.

## Conclusion

Germline variants play a role in 8.5%–10% of all pediatric cancer with the prevalence of certain genes such as *TP53*, *APC*, *NF1*, *PMS2*, *RB1*, and *RUNX1*. The increasing implementation and availability of genetic testing lead to the opportunity to identify the risk of cancer and early detection of tumors with the aim of reducing mortality and morbidity (21).

RTPS is characterized by a high risk of developing RTs and other unfrequent conditions. RTs are a rare, aggressive form of malignancies typically diagnosed in young infants that can arise in multiple anatomical sites. About 25%–35% of RTs carry a germline variant of *SMARCB1* (4, 5), or more rarely *SMARCA4*. The diagnosis of RTPS should be taken into account in patients with RTs, especially if early and multiple primary tumors and/or if a positive family history of RTs is present (25).

The ongoing new characterization of AT/RTs and RTs (12) will likely lead to further biological insights that can delineate molecular subtypes and may lead to novel therapeutic options. Despite these promising advancements, surveillance for cancer risk and prevention remains the focus of current management. Further research is needed to increase our understanding of RTs biology and gather further knowledge of the role of SMARCB1/SMARCA4 in RTs development and other rare manifestations.

## Author Contributions

GB and RC wrote the manuscript. PM provided the figure. AS, IA, and GM contributed to the finishing of the work. EA and MR contributed to the genetic details of the manuscript. AM, AC, LB, and FL revised it critically for important intellectual content. All authors contributed to the article and approved the submitted version.

## Conflict of Interest

The authors declare that the research was conducted in the absence of any commercial or financial relationships that could be construed as a potential conflict of interest.
